# Using anti-platelet therapy to prevent extracorporeal membrane oxygenator thrombosis without heparin resistance and with thrombocytopenia

**DOI:** 10.1186/s13054-014-0595-9

**Published:** 2014-10-30

**Authors:** Huai-Wu He, Xiang Zhou, Yun Long, Xiao-ting Wang, Qing Zhang, Hua Zhao, Hong-min Zhang, Wen-zhao Chai, Da-Wei Liu

**Affiliations:** Department of Critical Care Medicine, Peking Union Medical College Hospital, Chinese Academy of Medical Sciences, 1 shuaifuyuan, Dongcheng District, 100730 Beijing, China

Anticoagulation is widely used for preventing membrane oxygenator thrombosis during extracorporeal membrane oxygenation (ECMO) therapy while anti-platelets are rarely used, especially in patients with thrombocytopenia [1]. Here we report a case of successfully using anti-platelet treatment to prevent recurrent extracorporeal membrane oxygenator thrombosis in a fulminant myocarditis (FM) patient.

A 32-year-old man was admitted to our hospital with FM, with 10% left ventricular ejection fraction. After his arrival, he had a sudden onset of ventricular fibrillation and developed cardiac arrest, so cardiopulmonary resuscitation was performed. With high doses of vasoactive medications, he was transferred to the ICU, where venoarterial ECMO was initiated. Continuous infusion of heparin was used for anticoagulation, and activated partial thromboplastin time (aPTT) was titrated up to about 70 seconds. The rotational speed of the pump was about 4,500 rpm, and the blood flow of the pump was 3.9 L/minute. On the second day of ECMO therapy, the membrane oxygenator developed thrombosis, so we replaced it with a new one. Furthermore, we intensified the systemic anticoagulation with a higher aPTT target, about 70 to 90 seconds. However, the membrane oxygenator still developed thrombosis on the fourth day of ECMO initiation, and the oxygenator was replaced again. In addition, there was no clinical evidence of hemolysis or thrombosis at other body sites, and the platelet count decreased to 31,000/mm^3^. Thus, we inferred that the membrane oxygenator may have abnormally activated the platelets, resulting in thrombotic clot formation. As a result we combined the anti-platelet treatment (aspirin administered with a first dose of 300 mg and then 100 mg per day) with heparin therapy. After that, oxygenator thrombosis did not occur again, and the platelet count gradually increased to 123,000/mm^3^ without bleeding complications. The patient was weaned from ECMO 15 days after his admission. The related data for coagulation and anti-platelet treatment are summarized in Table 1.

**Table 1:** **Related data for anticoagulation and anti-platelet therapy during extracorporeal membrane oxygenation**

	**D1**	**D2**	**D3**	**D4**	**D5**	**D6**	**D7**	**D8**	**D9**	**D10**	**D11**	**D12**	**D13**
**Plt**	202	113	63	36	31	37	39	40	54	66	73	92	123
**aPTT**	77	68	91	97	65	58	56	61	60	79	77	74	64
**Aspirin**	-	-	-	300	100	100	100	100	100	100	100	100	100
Event		Ex-*		Ex-*									

D1 is the first day of extracorporeal membrane oxygenation initiation. Plt, platelet count (×10^3^/ml^3^); Aspirin, aspirin dose (mg); aPTT, activated partial thromboplastin time (seconds); Ex-*, oxygenator exchanged.

Ranucci and colleagues [2] reported that inadequate thrombin suppression by heparin may induce intravascular and extravascular thrombosis, which would result in coagulation factors and platelet consumption, and cause more bleeding and require larger transfusions. However, there was no evidence of insufficient anticoagulation and heparin resistance in our case presented here. Moreover, a higher target of anticoagulation was not effective for preventing oxygenator thrombosis, and the recurrent thrombosis happened only in the oxygenator but not at other sites of the body. Therefore, it seemed that the combination of anti-platelet treatment would be appropriate. However, thrombopenia is another dilemma. In our opinion, the primary cause of oxygenator thrombosis and thrombopenia was abnormal activation of the platelets in the membrane oxygenator, which deserved medical intervention. Lehot and colleagues [3] recently showed that oxygenator thrombosis occurred without heparin resistance in coronary artery bypass graft surgery with normothermic extracorporeal circulation in polycytemia vera. To the best of our knowledge, this is the first case report of the use of anti-platelet therapy to prevent oxygenator thrombosis without heparin resistance and with thrombopenia in FM.

## Abbreviations

aPTT: activated partial thromboplastin time
ECMO: extracorporeal membrane oxygenation
FM: fulminant myocarditis
